# The gut-brain axis: Effect of electroacupuncture pretreatment on learning, memory, and JNK signaling in D-galactose-induced AD-like rats

**DOI:** 10.22038/IJBMS.2023.66954.14683

**Published:** 2023

**Authors:** Min Xiao, Xue-Song Wang, Chuan He, Zhong-Sheng Huang, Hong-Ru Chen, Li-Hong Kong

**Affiliations:** 1 College of Acupuncture and Orthopedics, Hubei University of Chinese Medicine, Wuhan, Hubei, China; 2 Hubei Provincial Collaborative Innovation Center of Preventive Treatment by Acupuncture and Moxibustion, Hubei University of Chinese Medicine, Wuhan, Hubei, China; 3 Medical Department,Wuhan Red Cross Hospital, Wuhan, Hubei, China; 4 School of Acupuncture-Moxibustion and Tuina, Hebei University of Chinese Medicine, Shijiazhuang, Hebei, China; # These authors contributed equally to this work

**Keywords:** 5-HT, 5-HT6, Alzheimer’s disease, Brain-gut axis, Electroacupuncture – pretreatment, Gut microbiota, JNK signaling

## Abstract

**Objective(s):**

To examine the effect and potential mechanism of electroacupuncture (EA) pretreatment in spatial learning, memory, gut microbiota, and JNK signaling in D-galactose-induced AD-like rats.

**Materials and Methods:**

The AD-like rat model was generated by intraperitoneal injection of D-galactose. Morris water maze was used to determine spatial learning and memory ability, Real-time PCR to determine intestinal flora levels, ELISA to determine tryptophan (Trp) and 5-HT levels in the colon and hippocampal tissues, immunofluorescence to determine 5-HT levels in enterochromaffin cells (ECs), and immunoblotting to determine JNK signaling protein levels in hippocampal tissues.

**Results:**

Electroacupuncture pretreatment significantly reduced escape latency and prolonged exploration time in the target quadrant, and significantly increased the relative DNA abundance of *Lactobacillus* and *Bifidobacterium*. Meanwhile, electroacupuncture pretreatment also reduced colonic 5-HT levels and increased hippocampal 5-HT levels. Moreover, electroacupuncture pretreatment significantly inhibited hippocampal JNK pathway-related protein expression, including 5-HT6R, JNK, p-JUNK, c-JUN, and p-c-Jun. And the combination of GV20 and ST36 was more effective than single acupoints.

**Conclusion:**

Electroacupuncture pretreatment improved the learning and memory ability of D-galactose-induced AD-like model rats, changed the gut microbiota composition, and the mechanism may be related to the gut-brain axis and the JNK signaling pathway. In addition, the combination of GV20 and ST36 could further enhance the efficacy.

## Introduction

Alzheimer’s disease (AD) is a common degenerative disorder of the central nervous system (CNS) characterized by memory impairment, cognitive dysfunction, and personality and behavioral changes. Based on the World Alzheimer Report 2021, the number of AD patients is rising annually as global aging accelerates, with estimated 1 million new cases per year by 2050 (1). In addition, the number of AD- and dementia-related deaths has markedly increased during the COVID-19 pandemic (2, 3), making AD the sixth cause of death in the United States. Several factors influence AD progression, and age is the non-genetic factor most associated with AD. It has been shown that the incidence of AD doubles every 10 years after an individual reaches 60 years of age (4). Besides age, gender, vascular factors (e.g., Cardiovascular and cerebrovascular diseases, hypertension, and hyperlipidemia), and poor lifestyle are also essential factors that impact AD onset (5). The pathogenesis of AD is complex, and none of the current AD medications can reverse its progression. Recently, several clinical trials of new drug treatments for AD have ended in failure (6), possibly due to the tardiness of intervention and the advanced stage of the disease. Researchers have proposed early intervention as a prophylactic treatment (7), which may significantly delay AD progression, improve the quality of life of patients, and reduce the social and economic burden.

The human intestinal tract is a diverse and vibrant micro-ecosystem harboring 100 trillion microbial cells belonging to 1000–7000 species that are primarily grouped into anaerobes, facultative anaerobes, and aerobes (8, 9). Aging often accompanies reduced gut microbiota (GM) diversity (10-12). In addition, aging induces GM dysbiosis, leading to increased intestinal permeability. This, in turn, allows GM and its metabolites to enter the blood circulation, resulting in systemic inflammation (13, 14). Furthermore, GM also influences blood-brain-barrier (BBB) functions and basic neurodevelopmental processes, modulates brain functions (15), and causes cognitive impairment (16) via the gut-brain axis. Increasing evidence demonstrates that GM dysbiosis is associated with AD (17). In China, we have a long history of using acupuncture for the treatment of brain and gastrointestinal diseases, and plenty of clinical and experimental evidence has shown the potential of acupuncture in the treatment of dementia (18), Parkinson’s disease (19), depression (20), irritable bowel syndrome (21), functional dyspepsia (22), and gut dysbiosis (23). Our early works have shown that electroacupuncture (EA) can improve the learning and memory of AD rats, possibly through the regulation of the GM, inflammatory mediators, and microglia activation via the gut-brain axis (24, 25). In this study, we continued to examine the impact of EA on learning, memory, and JNK signaling in AD-like rats. 

## Materials and Methods

***Experimental animals and grouping***

Sixty SPF SD male rats (3–4 months, 360±20g) were purchased by the Hubei Experiment Animal Research Center (experimental animal production license: SCXK (Hubei) 2015-0018). The animals were housed at 20±2 °C with 40%–60% relative humidity, 12 hr/12 hr light/dark cycle, and access to food *ad libitum*. Rats were acclimatized for one week and randomized into the blank control group, model group, Zusanli (ST36) electroacupuncture (EA) pretreatment (EA-ST36) group, Baihui (GV20) EA pretreatment (EA-GV20) group, and the ST36 + GV20 EA pretreatment (EA-ST36+GV20) group, with 12 rats per group. All the animal experiments were conducted with the approval of the Ethics Committee of the Hubei University of Chinese Medicine.

***Model construction and intervention***

After a week of acclimatization, rats in all the groups except the blank control group were intraperitoneally injected with D-galactose (120 mg/kg) for eight consecutive weeks to induce AD (26-28). The specific methods are detailed in our previous studies (24, 29). Rats in all the EA groups received EA after D-galactose injection every day for eight consecutive weeks. The Baihui acupoint (GV20) is located in the center of the parietal bone, and the Zusanli acupoint (ST36) is situated in a depression 4 mm lateral to the anterior tibial node. EA was performed through disposable sterile acupuncture needles (0.30*15 mm; Beijing Zhongyan Taihe Medical Instrument Co., Ltd., China) that were horizontally inserted 3–5 mm into GV20 and vertically inserted 4 mm into both ST36. The positive electrode of the HANS electronic acupuncture apparatus (HANS-100A; Beijing Huayun Ante Science and Technology Co., Ltd., Beijing, China) was then connected to the needle at GV20, and the negative electrode was attached to ST36 (alternating between both the ST36). Based on our previous work (24), we applied continuous waves at a frequency of 50 Hz and a current of 1 mA as local vibrations at the acupoints. The animals were treated for 20 min once per day for eight weeks. Although the intervention was not given in the control and model groups, rats in these groups were restrained and fixed on a surgery board having the same dimension at the same time every day (once per day, 20 min each). 

***Observation parameters and test methods***

*Morris water maze test*

The water maze test was conducted after completing the intervention. The Morris water maze is a circular pool measuring 120 cm in diameter, 60 cm in height, and 30 cm in depth, with an 8 cm-diameter circular platform within quadrant 4. The pool was filled with water until the surface water was about 2 cm above the platform, and the water was maintained at 23±0.5 °C. Milk powder was added to turn the pool water milky white. The pool was divided into four quadrants, and one entry point was marked at the center of the pool edge of each quadrant. Navigation experiment: Rats were sequentially placed into the water facing the pool wall at the four entry points. The time required by the rat to find the platform (escape latency) each time was recorded. If a rat could not find the platform within 120 sec, the animal was guided to the platform for a 10 sec pause, and the escape latency was recorded as 120 sec. Rats in each group were continuously trained for 5 days. Spatial exploration experiment: the platform was removed on day 6. Rats were placed into the water at the quadrant three entry point, facing the pool wall and allowed to swim freely for 120 sec. The time it took the animal to pass the quadrant within which the platform was originally located was recorded.

*Rat tissue collection*

Upon completing the water maze test, rats were fasted for 12 hr for sample collection. Nine rats were randomly selected from each group and anesthetized by intraperitoneal injection of 1% pentobarbital sodium (4 ml/kg). Once the rats were fully anesthetized, the abdomen was incised, and the colon was collected into a labeled EP tube and flash-frozen in liquid nitrogen (n=6). Rats were subsequently euthanized by cervical dislocation, and the hippocampus was contained inside a labeled EP tube and flash-frozen in liquid nitrogen (n=6). The remaining three rats were anesthetized by intraperitoneal injection of 1% pentobarbital sodium (4 ml/kg) and placed in a supine position with all four limbs secured on a surgical platform. The chest was incised to expose the heart, and the perfusion needle was inserted within the left ventricle through the apex. The right atrial appendage was incised with scissors, and 0.9% sodium chloride solution was rapidly injected to flush the blood until the liver became off-white. Then, the animal was perfused and fixed with 4% paraformaldehyde, and the colon and brain tissues were collected in specimen tubes containing 10 volumes of 4% paraformaldehyde. The tubes were labeled and stored at 4 °C. The tissues were fixed for 72 hr and were cut into sections for subsequent histopathology.

*Real-time PCR*

Three rats were randomly selected from each group, and the bacterial DNA was extracted from mucosal scraping through centrifugation at 12000 r for 1 min using the Tissue Genomic DNA Extraction Kit (EP007, ELK Biotechnology, Wuhan, China). Once the RNA concentration and purity were determined, 2 μg of RNA was reverse transcribed using the cDNA synthesis kit (EQ003, ELK Biotechnology, Wuhan, China). The resulting cDNA was amplified by RT-PCR (StepOne^TM^ Real-Time PCR, Life Technologies). The primer sequences for *Lactobacillus*, *Bifidobacterium*, *Streptococcus*, and *Enterococcus* detection are shown in Table 1.

*ELISA*

Three additional rats were selected from each group, and Trp and 5-HT levels in the colon and hippocampal tissues were determined using the tryptophan (Trp) kit (catalog no. Ml059190, Shanghai Enzyme-Linked Biotechnology Co., Ltd.) and the 5-HT kit (catalog no. Ml028308, Shanghai Enzyme-Linked Biotechnology Co, Ltd), respectively. ELISA was performed as per the kit instructions, and the optical density (OD) of each well was measured at 450 nm using a microplate reader (USCNK, SMR60047). A standard curve was generated using the concentration and OD of the standard, and the sample concentration was then evaluated based on this standard curve.

*Immunoblotting*

Fresh frozen hippocampal tissues were selected from three rats in each group to detect JNK-related protein expression using Western blot. Tissue blocks were rinsed 2–3 times with pre-cooled PBS, cut into smaller blocks, and placed in a homogenizer. The tissues were homogenized in the RIPA lysis buffer (catalog no. AS1004, ASPEN, China) on an ice bath and centrifugated (12000×g at 4 °C for 5 min) to obtain the hippocampal tissue lysate. Protein concentration in the tissue lysate was determined using the Bicinchoninic Acid protein assay kit (catalog no. AS1086, ASPEN, China). An equal amount of the protein (40 μg) was loaded and separated in a 7.5–12% SDS-PAGE and transferred onto a polyvinylidene difluoride (PVDF) membrane (catalog no. IPVH00010, Millipore, Bedford, MA, USA). The membrane was blocked with 5% skim milk in Tris buffer containing 0.1% Tween-20 (TBS-T) at room temperature for 1 hr and then incubated using anti-5-HT6R (1:500, catalog no. bs-12058R, Bioss), anti-JNK (1:2000, catalog no. 9252, CST), anti-P-JNK (1:1000, catalog no. 4668, CST), anti-c-JUN (1:1000, catalog no. Ab32137, Abcam), and anti-P-c-JUn (1:1000, catalog no. 2361, CST) primary antibodies overnight at 4 °C. On the next day, the PVDF membrane was washed three times with TBS-T, incubated with HRP-goat anti-rabbit IgG secondary antibody (1:10000, catalog no. AS1107, ASPEN) for 30 min, incubated with freshly prepared ECL mixture (catalog no. AS1059, ASPEN), and visualized in a dark room.

*Immunofluorescence*

Immunofluorescence (IF) staining was performed on the perfused colon tissues of three randomly selected rats from each group to measure the 5-HT level in the enterochromaffin cells (ECs). The perfused and fixed rat colon tissue blocks were dehydrated, embedded in paraffin, cut into sections (about 5 μm thick), and incubated with anti-5-HT primary antibody (1:400, catalog no. Ab66047, Abcam) overnight at 4 °C. The tissue sections were washed, incubated with cyanine 3 (CY3, 1:50, catalog no. AS1113, ASPEN), washed three times, and stained using 4’,6-diamidino-2-phenylindole (DAPI). A few drops of the anti-fluorescence quenching agent were added to the tissue sections, and coverslips were mounted. Images of the tissue sections were acquired through a confocal laser scanning microscope (Leica, TCS SP8, Mannheim, Germany).

***Statistical analysis***

Data were expressed as mean ± standard deviation (mean ± SD) and analyzed using the GraphPad Prism 9 software. Data in each group were tested for normality and homogeneity of variance. Escape latency data from the Morris water maze navigation experiment were analyzed using two-way ANOVA, while all the other data were analyzed with one-way ANOVA. In addition, multiple comparisons were performed for each group using the SNK-q test. A *P*<0.05 was considered statistically significant.

**Table 1:** Primer sequences for gut microbiota detection

**Primer name**	**Sequence (** **5’-3’** **)**		
Lactobacillus	sense	GCAGCAGTAGGGAATCTTCCA	
	antisense	GCATTYCACCGCTACACATG	
Bifidobacterium	sense	GATTCTGGCTCAGGATGAACGC	
	antisense	CTGATAGGACGCGACCCCAT	
Streptococcus	sense	GTACAGTTGCTTCAGGACGTATC
	antisense	ACGTTCGATTTCATCACGTTG
Enterococcus	sense	TTGGCATTCCACAAGTACCA
	antisense	AATTGCTCGGGCATCATAAC

**Figure 1:**
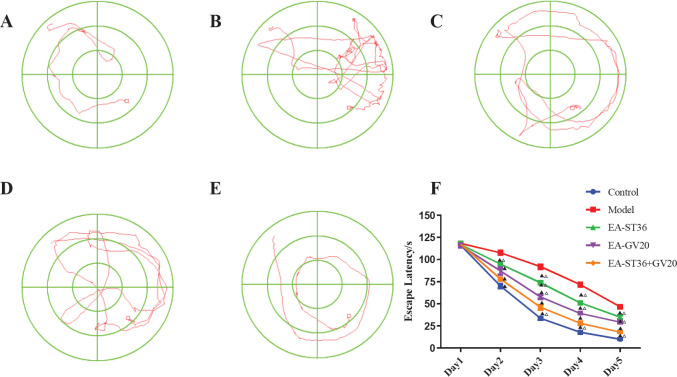
Comparison of escape latency among groups at different time points after the intervention. (A) Normal group; (B) Model group; (C) EA-GV20 group; (D) EA-ST36 group; (E) EV-GV20+ST36 group; The data are expressed as mean±SD (n=6). Compared with the model group, *P*<0.05; compared with the EV-GV20+ST36 group, *P*<0.05

**Figure 2:**
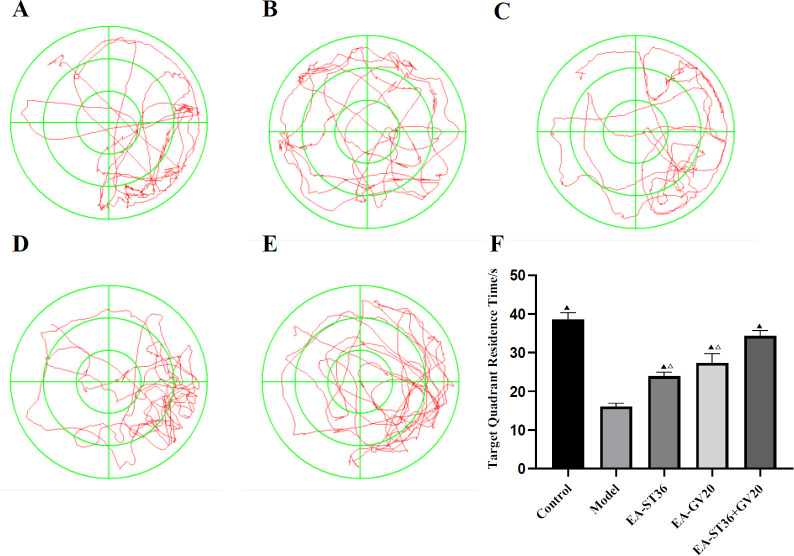
Comparison of retention time in target quadrant among groups after intervention in the spatial exploration experiment. (A) Normal group; (B) Model group; (C) EA-GV20 group; (D) EA-ST36 group; (E) EV-GV20+ST36 group. The data are expressed as mean±SD (n=6). Compared with the model group, *P*<0.05; compared with the EV-GV20+ST36 group, *P*<0.05

**Figure 3:**
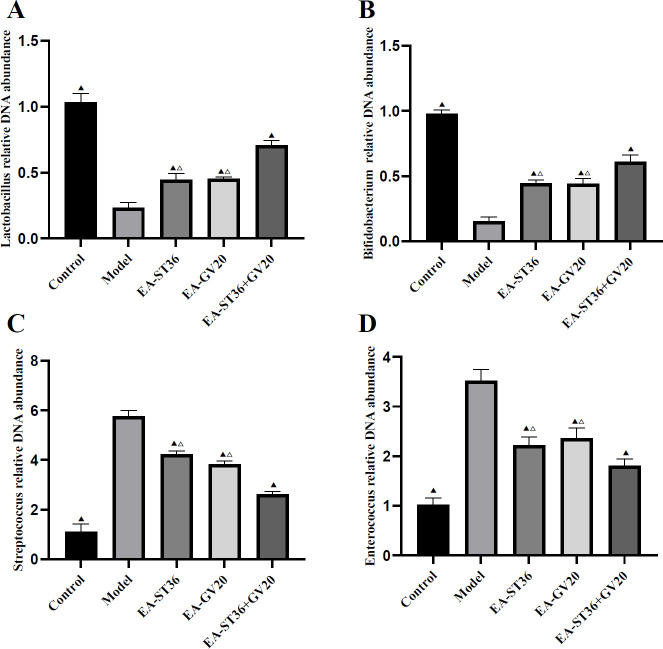
Relative DNA abundance of *Lactobacillus*, *Bifidobacterium*, *Streptococcus*, and *Enterococcus* DNA concentrations in rat colon. The data are expressed as mean±SD (n=3). Compared with the model group, *P*<0.05; compared with the EV-GV20+ST36 group, *P*<0.05

**Figure 4:**
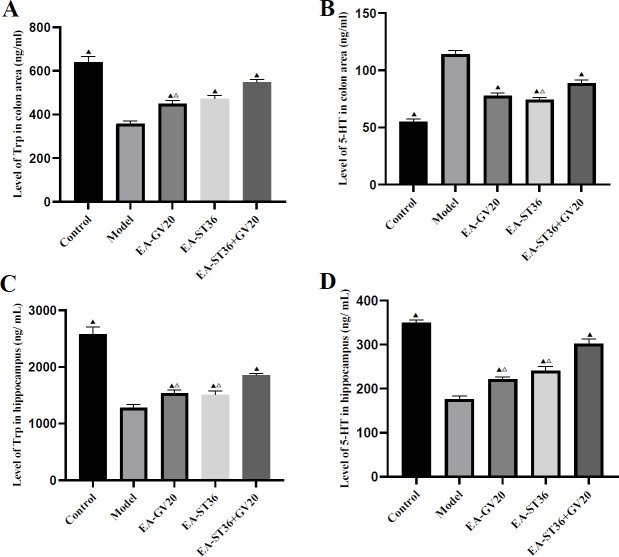
Colonic and hippocampal Trp and 5-HT levels. The data are expressed as mean±SD (n=6). Compared with the model group, *P*<0.05; compared with the EV-GV20+ST36 group, *P*<0.05

**Figure 5:**
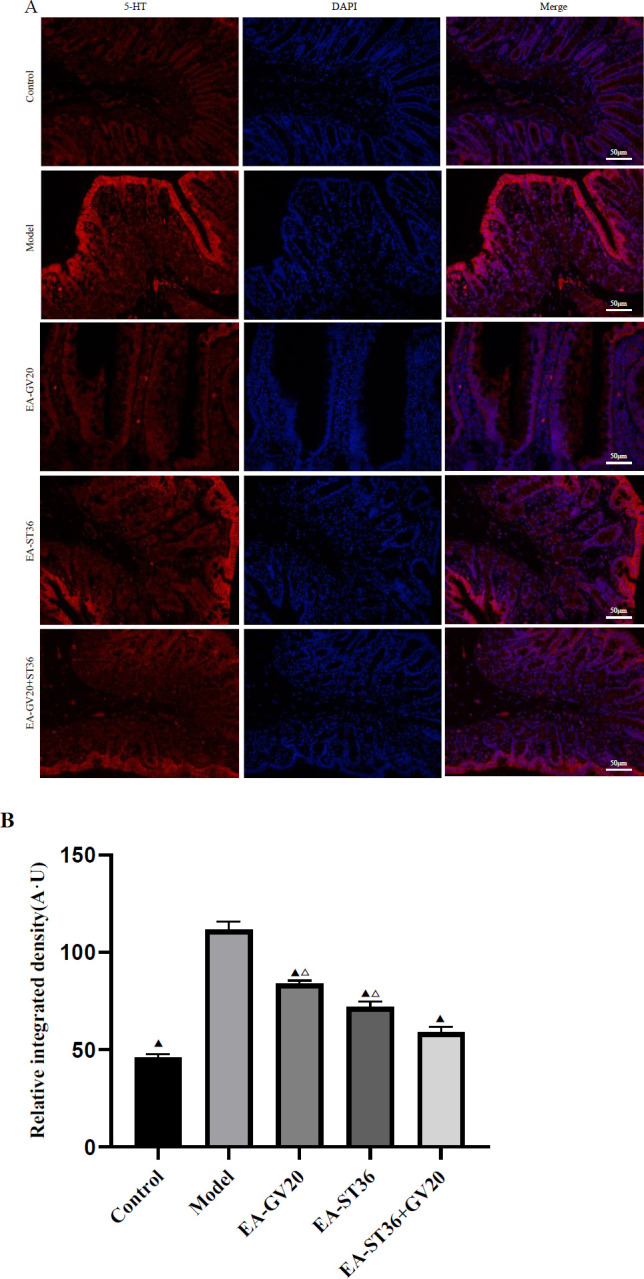
IF staining of 5-HT in ECs. (A) Micrograph of ECs in the colonic region, Magnification: 200 x, scale bar: 50 μm. (B) Quantitative photomicrograph analysis of the relative integrated density of 5-HT. The data are expressed as mean±SD (n=3). Compared with the model group, *P*<0.05; compared with the EV-GV20+ST36 group, *P*<0.05

**Figure 6:**
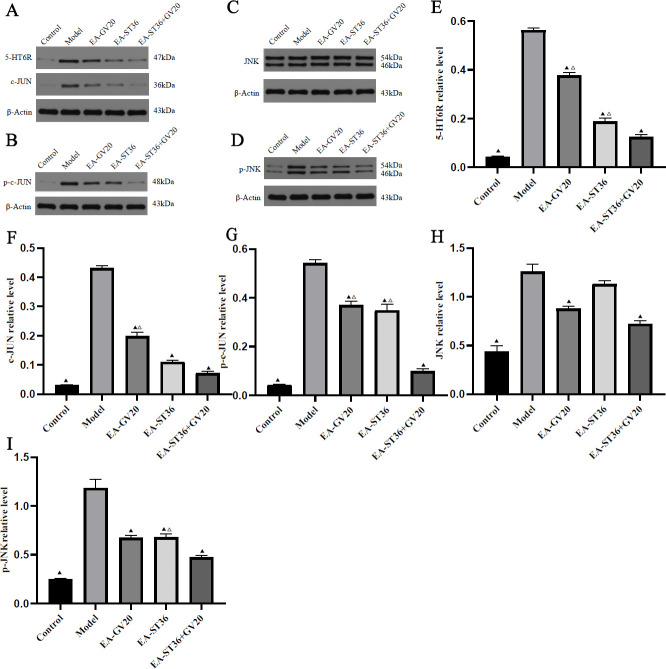
Regulatory effect of EA pretreatment on hippocampal JNK signaling. (A-I) Western blotting was used to detect the protein levels of 5-HT6R, c-Jun, p-c-Jun, JUK, and p-JUK in the hippocampus. The data are expressed as mean±SD (n=3). Compared with the model group, *P*<0.05; compared with the EV-GV20+ST36 group, *P*<0.05

## Results

***Morris water maze***

On day 1 of the navigation experiment, rats from each group primarily swam beside the pool wall and were rarely near the platform. The motion trajectory of the animals was randomly distributed among the four quadrants. Starting on day 2, the time required by each group of rats to find the location of the platform using spatial cues gradually decreased, indicating a downward trend. In addition, the swimming trajectory of the rats in the target quadrant became more concentrated. As shown in Figure 1, the escape latency of rats was significantly prolonged in the model group versus the normal group (*P*<0.05). However, it was significantly shortened after intervention in the EA-ST36, EA-GV20, and EA-ST36+GV20 groups versus the model group (all* P*<0.05), which indicated that EA pretreatment could improve spatial learning and memory impairment in D-galactose-induced AD-like rats. In addition, escape latency was significantly shorter in the EA-ST36+GV20 group than in EA-ST36 and EA-GV20 groups (*P*<0.05), demonstrating that EA-ST36+GV20 was superior to EA-ST36 and EA-GV20 alone in preventing spatial learning and memory impairment within D-galactose-induced AD-like rats.

The spatial exploration experiment was conducted six days after the completion of the navigation experiment. As shown in Figure 2, exploration time in the target quadrant was significantly lower in the model group than in the normal group (*P*<0.05). In contrast, exploration time was significantly prolonged in the EA-GV20, EA-ST36, and EA-GV20+ST36 groups versus the model group (all *P*<0.05). The motion trajectory was primarily concentrated around the target platform, indicating that EA pretreatment improved the spatial learning and memory of D-galactose-induced AD-like rats. Furthermore, EA-GV20+ST36 showed a better protective effect on learning and memory than EA-GV20 and EA-ST36 alone (*P*<0.05).

***Colonic microbiota of each group***

As shown in Figure 3, compared with the normal group, the model group had significantly reduced abundances of *Lactobacillus* and *Bifidobacterium* (*P*<0.05) and significantly increased abundances of *Streptococcus* and *Enterococcus* (*P*<0.05). After the intervention, the abundances of *Lactobacillus* and *Bifidobacterium* were significantly higher. The abundances of *Streptococcus* and *Enterococcus* were significantly lower in the EA-GV20, EA-ST36, and EA-GV20+ST36 groups versus the model group (all *P*<0.05). Furthermore, EA-GV20+ST36 had a better regulatory effect on GM than EA-GV20 and EA-ST36 alone (*P*<0.05).

***Colonic and hippocampal Trp and 5-HT levels***

Colonic Trp, hippocampal Trp, and hippocampal 5-HT levels were significantly lower, while the colonic 5-HT level was significantly higher in the model group than in the normal group (all *P*<0.05). After the intervention, colonic Trp, hippocampal Trp, and hippocampal 5-HT levels were significantly higher. In contrast, the colonic 5-HT level was significantly lower in the EA-GV20, EA-ST36, and EA-GV20+ST36 groups versus the model group (all *P*<0.05). In addition, EA-GV20+ST36 had a better regulatory effect on colonic and hippocampal Trp and 5-HT levels than EA-GV20 and EA-ST36 alone (all *P*<0.05) (Figure 4).

***IF staining of 5-HT in rat ECs***

As shown in Figure 5, 5-HT level in ECs was significantly higher in the model group than in the normal group (*P*<0.05).. However, 5-HT level in ECs was significantly lower in the EA-GV20, EA-ST36, and EA-GV20+ST36 groups than in the model group (all *P*<0.05), indicating that EA pretreatment could down-regulate 5-HT level in ECs. Moreover, EA-GV20+ST36 had a better regulatory effect on 5-HT levels in ECs than EA-GV20 and EA-ST36 alone (*P*<0.05).

***Expression of JNK pathway-related proteins in rat hippocampus***

As shown in Figure 6, compared with the normal group, the model group had significantly up-regulated hippocampal 5-HT6R, JNK, p-JUNK, c-JUN, and p-c-Jun protein expression (*P*<0.05). Upon intervention, the EA-GV20, EA-ST36, and EA-GV20+ST36 groups showed significantly down-regulated hippocampal 5-HT6R, p-JNK, c-JUN, and p-c-Jun expression, and the EA-GV20 and EA-GV20+ST36 groups had significantly down-regulated hippocampal JNK expression compared with the model group (all *P*<0.05). In addition, EA-GV20+ST36 had a better regulatory effect on the expression of JNK pathway-related proteins in the hippocampus than EA-GV20 and EA-ST36 alone (*P*<0.05).

## Discussion

The incidence of AD is positively correlated with aging. As global aging accelerates, AD has become one of the most critical medical and societal problems in the world. AD has an insidious onset with an irreversible disease course, and no current medications or treatments could delay and improve AD. In this study, we used a rat model of D-galactose-induced aging to mimic the process of natural aging. D-galactose is a reducing sugar, and higher doses induce a series of responses that accelerate the aging process and are mostly used in aging-related research. In recent years, many studies have shown that D-galactose-induced aging models also have AD-like pathology (30, 31), including oxidative stress damage (32), neuroinflammation (33), dysregulation of APP metabolism (34), and phosphorylation of Aβ and Tau proteins(35). In our previous study, we also used this model and observed neuroinflammatory changes (24) and tau protein pathology in the rat brain (29, 36). Therefore, this study continues to use this AD-like pathology model, which aims to simulate the process of normal aging to AD-like pathology, which is in harmony with the traditional Chinese medicine theory of “preventive treatment for disease”, and reflects the importance of prevention, i.e. prevention is better than treatment. This study observed impaired spatial orientation and memory in the model group, consistent with previous studies. 

According to traditional Chinese medicine theory, the pathological changes in AD are in the brain, and GV20 belongs to the Governor Vessel, which is located at the top of the head and has the function of xingnao kaiqiao; and ST36 belongs to the foot Yangming Stomach meridian and has the effect of regulating the spleen and stomach. In addition, the results of a bibliometric study of acupoints used in the clinical treatment of AD also showed that both Baihui and Sansili were used more frequently in clinical practice (37). Therefore, we chose GV20 and ST36 as the acupoints for our study. Our results show that EA pretreatment markedly improved the cognitive functions of AD rats in the Morris water maze, and the efficacy of EA-GV20+ST36 was superior to that of EA-GV20 and EA-ST36 alone. Thus, it indicated that EA-GV20+ST36 is more effective in improving learning and memory impairment in AD rats.

The gut microbiota (GM) refers to the trillions of microbes that colonize the animal intestine and is an essential component of the intestinal micro-ecosystem. Under physiological and pathological conditions, GM can influence the functions and behaviors of the brain through bidirectional regulation of various immune, endocrine, and vagus nerve pathways via the gut-brain axis (38). GM is closely associated with the development and cognitive behaviors of the nervous system, and evidence indicates that GM is closely linked to AD pathogenesis (38, 39). This study used real-time PCR to measure the GM DNA level, including *Lactobacillus*, *Bifidobacterium*, *Streptococcus*, and *Enterococcus*. Our results showed that EA pretreatment significantly increased the abundances of *Lactobacillus* and *Bifidobacterium *and significantly reduced the abundances of *Streptococcus* and *Enterococcus* in AD rats. In addition, EA-GV20+ST36 depicted a more potent regulatory effect on GM, demonstrating that EA could improve cognitive impairment by influencing GM composition.

5-HT (serotonin) regulates various physiological processes, such as cognition, emotion, sleep, and food consumption. The 5-HT receptor is highly expressed in brain learning and memory regions (e.g., hippocampus, amygdala, and cortex) (40). Numerous studies (41, 42) have shown that aberrant 5-HT level plays a vital role in AD development and progression, including regulating pathological and clinical manifestations. The essential amino acid tryptophan (Trp) is the primary substrate for the synthesis of 5-HT. Trp is present in dietary proteins and is acquired by exogenous dietary intake in humans. It was reported that *Bifidobacterium *could increase plasma Trp levels to influence intracerebral 5-HT transmission (43). About 95% of 5-HT in the human body is generated inside the intestine and synthesized by the ECs and myenteric plexus. Under the influence of GM, ECs utilize tryptophan hydroxylase (TPH1) to synthesize 5-hydroxytryptophan (5-HTP), which is then converted to 5-HT by tryptophan decarboxylase (TDC). Therefore, GM is the main factor that influences peripheral 5-HT synthesis. Under normal conditions, Trp and 5-HTP, but not 5-HT, can enter the BBB from the periphery and indirectly regulate the 5-HT synthesis and pathways in the CNS. As a result, 5-HT is a crucial factor in the GM-gut-brain axis (44). We used ELISA to measure the changes in Trp and 5-HT levels in the colon and hippocampus of Ad rats. It was observed that EA intervention significantly up-regulated colonic Trp, hippocampal Trp, and hippocampal 5-HT levels but down-regulated colonic 5-HT levels. Similarly, IF staining of 5-HT in ECs showed that EA intervention reduced colonic 5-HT content in AD rats, and this effect was more potent in the EA-GV20+ST36 group. This finding indicated that EA could improve the cognitive functions of AD rats by influencing 5-HT synthesis and metabolic pathways via improvement of GM dysbiosis, which is consistent with previous studies.

The primary physiological functions of 5-HT are mediated through selective actions of 5-HT on different receptors. 5-HT6R is a subtype of 5-HT receptor exclusively expressed in the CNS, especially in the prefrontal cortex and hippocampus, associated with learning and memory, respectively (45). 5-HT6R is most closely related to cognition and has recently become a new drug target for improving cognitive functions (46). Studies have shown that rats with increased 5-HT6R levels in the dorsal medial striatum, cortex, and hippocampus have difficulty completing instrumental learning tasks. Therefore, it suggests that 5-HT6R up-regulation impairs learning and memory, and blocking 5-HT6R can treat cognitive dysfunction (45, 47). It was reported that 5-HT6R is involved in mediating intracellular JNK signaling. JNK activation increases phosphorylation, critical in several cell regulatory processes such as cell proliferation, differentiation, apoptosis, and stress response (48). It is involved in the development and progression of numerous diseases and pathological damages. In addition, the protein expression and phosphorylation of the downstream molecule c-Jun are also elevated, synergizing with JNK to induce neuronal apoptosis and affect the CNS (49). Cell apoptosis is an essential mechanism of AD pathogenesis (50). Our study showed that EA intervention significantly down-regulated 5-HT6R, JNK, p-JNK, c-JUN, and p-c-Jun expression in the hippocampus of AD rats. Moreover, the effect was better in the EA-GV20+ST36 group, indicating that EA-GV20+ST36 could improve learning and memory impairment in AD rats by down-regulating 5-HT6R levels and inhibiting JN K signaling.

## Conclusion

EA-GV20+ST36 can improve spatial learning and memory of AD rats, possibly by regulating GM, inhibiting hippocampal JNK signaling, and down-regulating hippocampal 5-HT6R, JNK, p-JNK, c-JUN, and p-c-JUN expression via the gut-brain axis. Given the complexity of AD pathogenesis, the possibility that the therapeutic effect of EA pretreatment may also be mediated through other signaling pathways cannot be excluded. Therefore, the molecular mechanism of EA in preventing and treating AD warrants further investigation.

## Authors’ Contributions

MX, XSW, and LHK designed the experiments; CH, ZSH, and HRC performed experiments and collected data; MX, XSW, and LHK discussed the results and strategy; LHK supervised, directed, and managed the study; MX, XSW, CH, ZSH, HRC, and LHK approved the final version to be published.

## Funding

This study was supported by the National Natural Science Foundation of China (No.81373741), “Qihuang” Project on Inheritance and Innovation of Traditional Chinese Medicine funded by the National Administration of Traditional Chinese Medicine.

## Conflicts of Interest

All authors declare that they have no potential conflicts of interest.
